# Impact of a Core Ferrule Design on Fracture Resistance of Teeth Restored with Cast Post and Core

**DOI:** 10.1155/2016/5073459

**Published:** 2016-06-22

**Authors:** Loubna Shamseddine, Farid Chaaban

**Affiliations:** ^1^Department of Prosthodontics, Lebanese University, School of Dentistry, P.O. Box 6573/14, Beirut, Lebanon; ^2^Faculty of Engineering and Architecture, American University of Beirut, Mazraa-Daybess Street, Ferdawss Building, First Floor, P.O. Box 11-0236, Beirut 1107 2020, Lebanon

## Abstract

*Objectives*. To investigate the influence of a contra bevel on the fracture resistance of teeth restored with cast post and core.* Materials and Methods*. Sixty plastic analogues of an upper incisor were endodontically treated and prepared with 6° internal taper and 2 mm of ferrule in order to receive a cast post and core. The prepared samples were divided into two groups (*n* = 30); the first group serves as control while the second group was prepared with an external 30° bevel on the buccal and lingual walls. All samples crowned were exposed to a compressive load at 130° to their long axis until fractures occurred. Fracture resistance loads were recorded and failure modes were also observed. Mann-Whitney test was carried out to compare the two groups.* Results*. Mean failure loads for the groups were, respectively, 1038.69 N (SD ±243.52 N) and 1078.89 N (SD ±352.21 N). Statistically, there was no significant difference between the two groups (*P* = 0.7675 > 0.05).* Conclusion*. In the presence of a ferrule and a crown in the anterior teeth, adding a secondary ferrule to the cast post and core will not increase the resistance to fracture.

## 1. Introduction

The prognosis of endodontically treated teeth (ETT) is proven to be affected by the type of the restoration [1, 2], and in this aspect numerous methods of restoring ETT have been advocated. The traditional approach for restoration of ETT with moderate-to-severe tooth loss is to make a post and core and, subsequently, place a crown [3, 4]. Present options include cast metal posts and cores and prefabricated metal or fiber-reinforced composite posts [5]. The purpose of the post is to retain coronal structure restoration with the ability to save severely damaged teeth. Cast posts and cores are considered as the restorative method of choice for anterior teeth with moderate and severe destruction [4, 6]. Custom cast post and core allow for a close adaptation of the post-to-post space preparation and should fit optimally [4].

Common failure types of ETT restored with cast posts vary from post dislodgment to root fracture. The latter is the primary reason for the extraction of such teeth [7]. In fact, ETT often have little coronal tissue remaining due to caries, trauma, cavity preparation, and/or root canal treatment, making them even more susceptible to fracture [8, 9].

Several factors affecting fracture strength of ETTs are found in the literature; some are related to the tooth restored and others to the type of post used. Tooth location is also one of these factors. In fact, the magnitude and direction of functional loads play a major role in stress concentration within the dowel-restored teeth [10]. Anterior teeth undergo nonaxial forces more than posterior teeth that are primarily axially loaded [11]. Nonaxial forces are more detrimental to the tooth restoration interface [8, 12] and increase the frequency of fracture [13]. The preparation of the tooth is another parameter directly related to the fracture resistance. An adequate resistance to displacement of every cast restoration depends largely on the retention and resistance form in the preparation [14]. The ideal taper recommended varies from 2° to 7° per axial wall. This taper is suggested to avoid forming undercuts to the withdrawal axis of a cast post [15, 16]. Clinically, the reported ideal axial wall convergence values for full coverage restorations are ranging from 4° to 20° [17, 18].

Different means as ferrule effect, interlocking devices, grooves, and contra bevel have been suggested to improve retention and enhance the resistance of ETT. Most recent studies agree that the most important factor of success when restoring ETTs with post and core is the ferrule. This encompassing band of cast metal around the coronal surface of the tooth may resist stresses such as functional lever forces, wedging effect of posts, and the lateral forces exerted during the post insertion [19]. To ensure durability, ETTs must have a ferrule height of at least 1.5 to 2 mm [20, 21]. It operates as an anti-rotary device and improves the biomechanical stability of the tooth [9, 22–25]. Ferrule design has been studied and found to produce greater strength when it is circumferential and uniform [26–28]. Various ferrule designs have been suggested but currently there is little research supporting one design over the others [29, 30].

The post type and its adaptation are the major factors affecting the strength of ETTs. Relevant reports and studies have indicated that cast posts are proven to have higher fracture resistance compared to fiber posts [31–33]. Similarly, fracture strength in the anterior teeth has been reported to be higher with cast posts than with fiber posts [34–36] and higher than that of prefabricated titanium post and composite core [37]. Although cast post and cores restored teeth showed higher prevalence of irreparable failures [38], they exhibit a high survival rate up to 19.5 years [39].

The main advantage of using a cast post and core technique is the ability to conform to any canal space and to provide a good fit that would lead to uniform distribution of forces within the root [37, 40]. Moreover, in cast post and core restorations, a balance exists between maximizing retention and maintaining resistance to root fracture [41]. Cast posts would fit passively into the canal and would resist rotation and rocking [41]. Grooves have been advocated as additional retention through the preparation as a means for improving the crown retention [42] with an antirotational mean for the post [14]. However, the incorporation of the antirotational device in cast post and core on the buccal and lingual faces concomitantly was found to increase the stress-strain values [43] but was judged to be insignificant in terms of fracture resistance of the teeth [43–45].

The contra bevel has also been suggested as a secondary ferrule and as an antirotational mechanism incorporated to the cast post and core [14, 28, 44, 46, 47]. It is an external bevel arising from the occlusal surface or edge of a tooth preparation and placed at an angle that opposes or contrasts the angle of the surface it arises from [48]. This contra bevel used as a core ferrule has been found to enhance fracture resistance in several studies [47, 49, 50], while in other studies no significant effects were observed [46, 51]. It should be noted that the fracture resistance studies did not take the full coverage crowns into consideration. An external 30 degrees bevel may be of interest for strengthening ETT because it acts as a positioning guide and as an antirotational device for the post and core.

Based on the positive correlation between the retention of the cast post and core and the resistance to fracture of the teeth, the antirotary resistance form, realized by the contra bevel, could modify the load direction and stress distribution within the post/dentin system.

The purpose of this study is to assess the effect of a contra bevel on the fracture resistance of crowned anterior teeth restored with cast posts and core. The null hypothesis being tested was that there is no difference between the two types of teeth prepared with or without a contra bevel.

## 2. Materials and Methods

**Figure 1:**
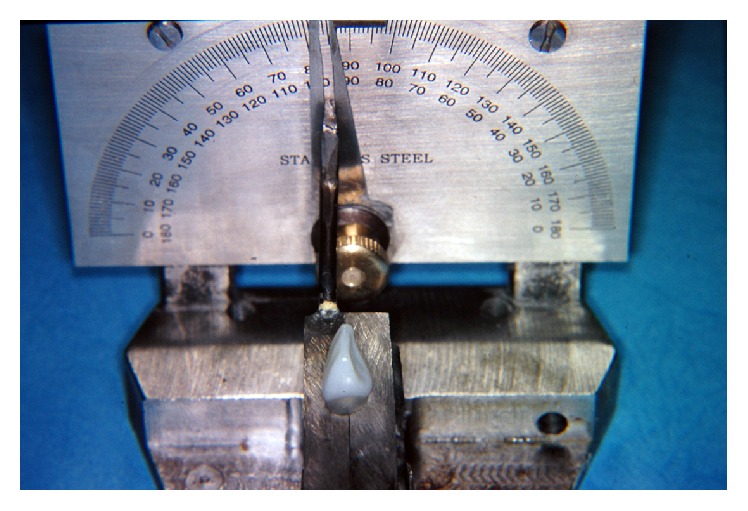
Metallic block with protractor used for the experiment.

Sixty clear acrylic standardized analogues B22X-500 (Kilgore International, Inc., USA) simulating ETT maxillary central incisors and a special metallic device were fabricated to subsequently mount the analogues for this experiment. This device was made of a base and a mounting block with a hinge access having the ability to move in mesiodistal direction thus allowing the axis of analogues to be fixed with different rotational angles. In addition, a protractor instrument related to the base was used for the inclination of the block (Figure 1).

### 2.1. Preparation of the Crown

**Figure 2:**
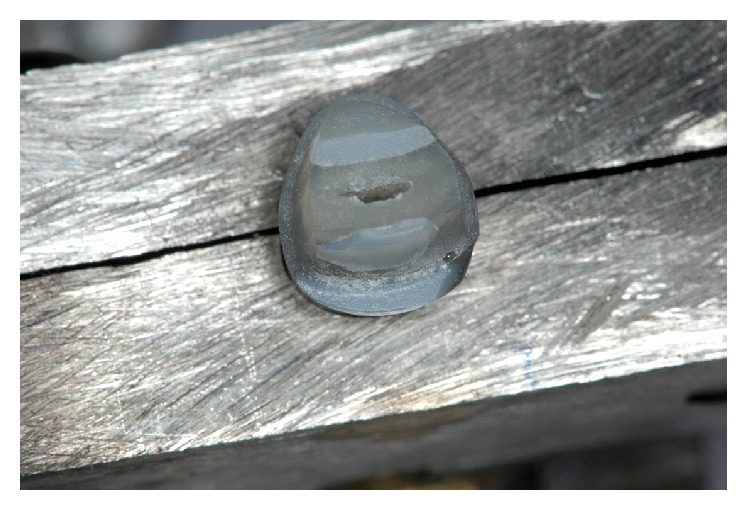
Coronal preparation for full coverage crown in both groups.

**Figure 3:**
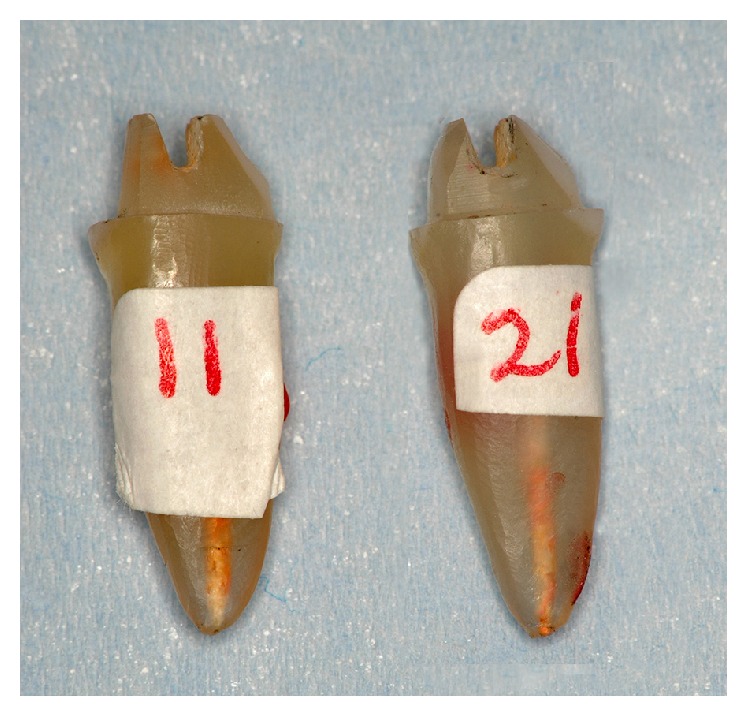
Specimen of each group showing the prepared contra bevel in group 2.

Into the block, all specimens were prepared using an electronic surveyor. The crowns were prepared perpendicular to the root axis with an abrasive disc (X928-7 TP, Abrasive Technology, Inc., USA), leaving 5 mm above the cervical area. The axial surfaces of the tooth were prepared with specific burs (facial: 1.5 mm ISO number 806, 104 173 544 031, palatal and proximal: 0.8 mm ISO number 806, 104 173 544 016) following the cement enamel junction to receive a full coverage metal crown (Figure 2). The specimens were divided into two groups of 30 analogues each. A 2 mm ferrule was left at the proximal sides. Group 1 was considered as control group without modifications, while group 2 was obtained by changing the axis of the analogues to 24° and the facial and palatal walls were prepared to create an external bevel 30° to the long axis of the tooth (Figure 3).

### 2.2. Endodontic Preparation

Analogues were prepared with an access cavity of 6° taper using the protractor at 0° and burs with 6° taper. Root canal preparation was then executed with the protaper system according to the manufacturer's instructions to a working length of 18 mm. Gutta-percha was then laterally condensed with a manual spreader (Kerr, W 0697840 and W 0693510).

### 2.3. Post and Cores Fabrication

**Figure 4:**
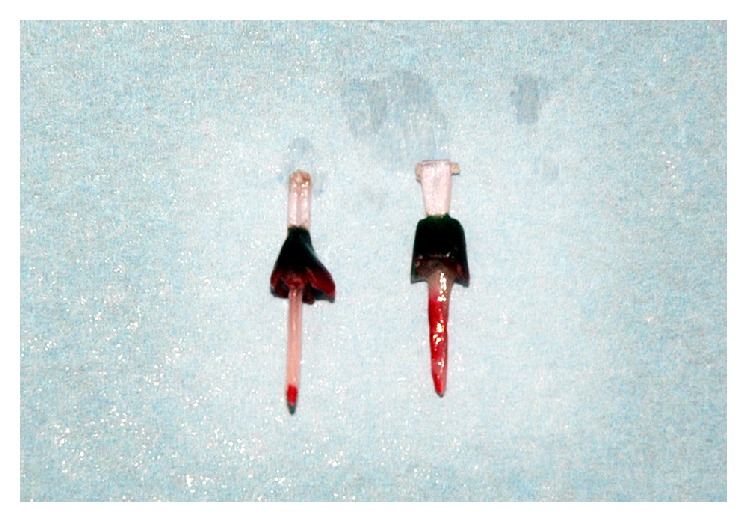
Specimens of post and core fabricated with wax patterns.

**Figure 5:**
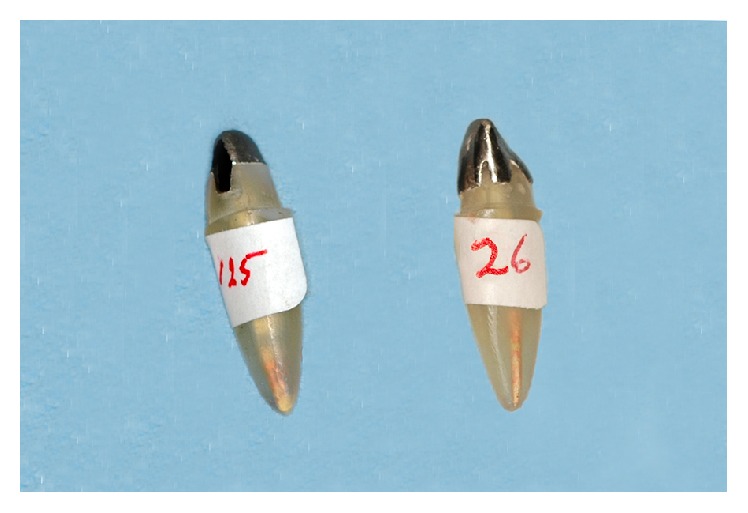
Specimen of cemented cast post in each group.

Gates Glidden drill number 3 (Dentsply Maillefer A0008 240 00500) and 1.1 mm Largo (Dentsply Maillefer A0008 230 002 00 to A0008 230 003 00) were used to prepare the post spaces, leaving 7 mm of apical seal. Post and cores were constructed using 1.25 mm burn-out plastic posts coated in increments with wax patterns to fit the root canal. For the coronal fabrication, a silicone index of the intact tooth and wax were used to standardize the coronal dimension for all specimens. The bevel at the coronal part in group 2 was filled by wax patterns thus becoming a part of the post and core (Figure 4). The post and cores were cast in a Ni-Cr alloy and cemented using spiral paste filler (Dentsply Maillefer Instrument) under a static load 1.5 Kg for a duration of 15 min with zinc phosphate cement (spofaDental Adhesor®) (Figure 5).

### 2.4. Cast Crowns Fabrication

**Figure 6:**
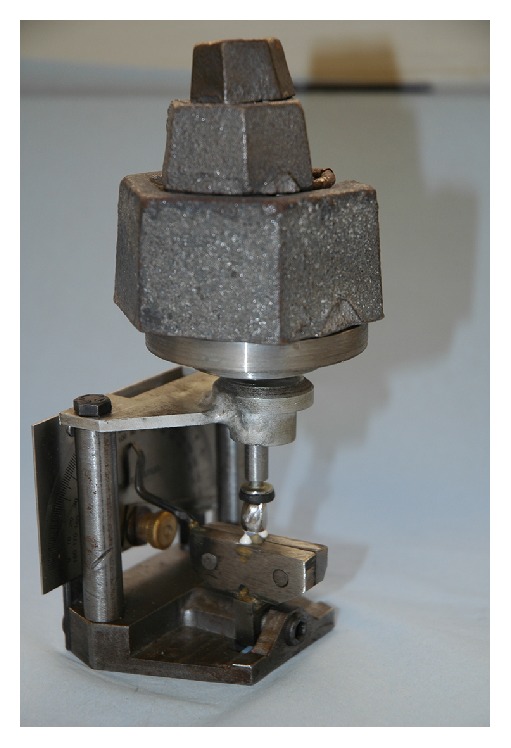
Cementation of a crown under static load.

After removing cement excess, a crown was waxed and adapted directly to the analogue using the previously prepared silicon index. After investing the wax pattern and casting it with Ni-Cr, the crown was cemented with zinc phosphate under a static load of 1.5 kg, also for 15 min (Figure 6).

### 2.5. Fracture Testing

**Figure 7:**
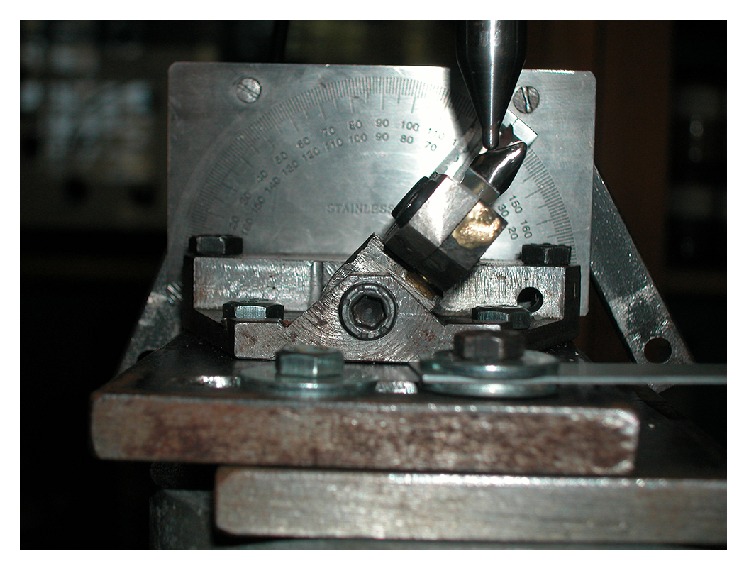
Simulating clinical direction in class I occlusion for testing.

Fracture strength testing was then performed on the two groups, in the laboratories of the Mechanical Engineering Department at the American University of Beirut, Lebanon. The testing device is a tension and compression system (YLUTM) and is fully computerized. This testing machine has an error margin of 0.04% for maximal load of 10 000 kg, a margin of 0.01% for repetitive maximal load of 10 000 kg with a resolution of displacement of 0.01 mm (10 *μ*m) and accurate speed of 0.01% of full scale. The crowned analogues were subjected to an inclined compressive load (with a 1-kN cell at a crosshead speed of 0.05 mm/min at 130° to the long axis) divided into a compressive and bending components until fracture occurred (Figure 7).

## 3. Statistical Analysis

Data from the test results were analyzed using statistical software (SPSS 17; SPSS Inc., USA) and, for each group, load to fracture mean values and standard deviations (SD) were calculated.

Lilliefors test was used to check for normality and subsequently the Mann-Whitney test was used to compare fracture resistance between the groups. For the displacement values, the Kruskal-Wallis test was used to compare the groups. The level of significance (*P*) was set at 0.05.

## 4. Results

**Figure 8:**
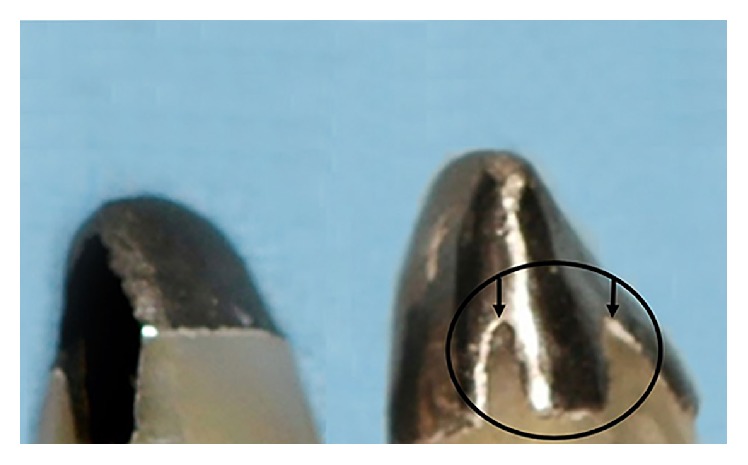
Illustration of the incomplete seating of post and cores in group 2.

**Figure 9:**
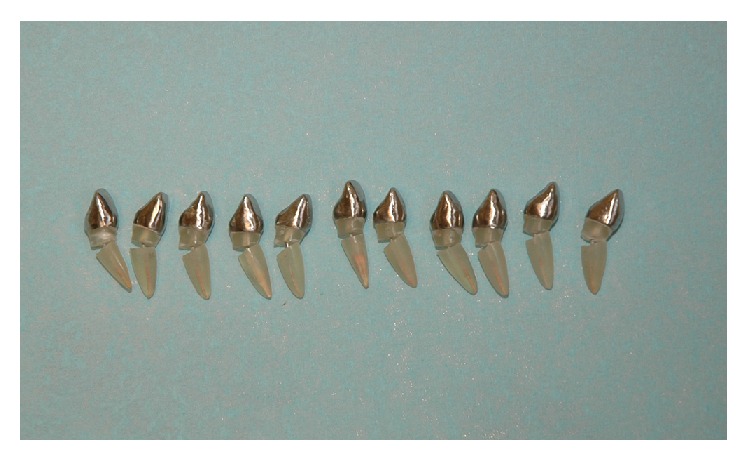
Fracture line visualized after testing in all specimens.

Incomplete seating of posts for all specimens of group 2 was the first result noticed before fracture testing (Figure 8). Another finding is that all analogues failed with the same line direction and level after fracture testing (Figure 9). Considering the values obtained, the same displacement was observed for both groups. Statistical results are summarized in Table 1. There was no significant difference between the two groups for fracture load (*P* = 0.7675) as well as for the displacement (*P* = 0.7470). Means and standard deviations for the two parameters are also given in Table 1.

## 5. Discussion

Since fracture load depends on the geometry of teeth [52], this study used acrylic analogue to compare between the tested specimens as human incisors would have had a larger variability in size and morphology. This variability would have been otherwise required to observe significant differences between the two groups.

The localization of fracture lines for all specimens obtained in this study is attributed to the metallic device that holds the specimens during the testing process. The level and direction in the mouth could be different since bone and periodontal ligaments affect the strength of the roots [53, 54]. This metallic device explains the result of almost no difference found in the displacement between the two groups. The major objective of this study was to find which preparation design had a better resistance force to fracture, despite the load values or the failure localization.

The hypothesis that the mechanical behavior of anterior endodontically treated teeth would be affected by the ferrule added to the cast post and core was rejected. A slight increase in the fracture load has been found in group 2 without a significant difference in group 1 (*P* value = 0.7675).

The results of the present study indicate that a contra bevel incorporated to the custom cast post core did not improve the fracture resistance of ETT. The results of the study are in agreement with previous studies conducted by Sorensen and Engelman in 1990, Kutesa-Mutebi and Osman in 2004, and by Goyal et al. in 2007 [29, 46, 51].

It was stated that as the volume of posts decreases, the absorption of forces by the post system also decreases to a considerable degree [55]. In the study, group 2 with a larger volume of cast post and core demonstrated an equivalent fracture strength compared to the smaller volume of group 1. This could be attributed to the inconvenience of extra coronal additional part and its casting simultaneously to the intracoronal part. In fact, casting an extracoronal restoration differs from that of the post and core. It is necessary to fabricate a slightly undersized cast post to allow for passive fit and cement placement [56, 57], while oversized castings could give a better adapted crown margin upon cementation than an undersized one [58]. The study was carried out to develop undersized cast post and cores to fit passively the shape of the post space, in order to lead to a better transmission of the stresses. The two groups should exhibit the same adaptation of the post into the canal since they have the same root preparation, same post and core fabrication, and same cementation protocols. The similar fracture strength found in both groups could be explained by the cementation technique used in both groups (static load of 1.5 kg for a duration of 15 min) and especially to the equivalent taper of the canal and cavity walls. However, the undersized contra bevel makes it more difficult for air and excess cement to escape from the canal thus increasing the occurring of the filtration phenomenon. This phenomenon could prevent the post from being well placed and could affect the physicochemical properties of the cements and biomechanical behavior of the fixed restauration [37] with a higher film thickness than the ADA spec number 8 Zinc phosphate cement [59]. A similar pattern in the group 2 is possible as extracoronal casted parts can lead to the incomplete seating of posts for all specimens. This finding is supported by Dreyer and Jørgensen 1955 and Dimashkieh et al. 1974 who found that a filtration phenomenon can occur in the cementation onto well-fitted teeth preparations using zinc phosphate cement. When the passage of cement is reduced and large grains of cement powder begin to jam together, cement liquid filtration occurs and this resulted in an uneven distribution of cement powder portion in the phosphate matrix. The solid particles would form a mass that allows passage of the thinner liquid only causing further separation and filtration of the cement [60, 61].

The absence of adequate relief spaces impedes the flow of cement, leading to incomplete seating because of hydraulic pressure [62]. Dreyer and Jørgensen suggested that when the crown carrying the cement is placed on the prepared tooth, cement accumulates on the occlusal surface [60] and when pressure is applied to complete the seating of the crown, the excess cement can escape only through the space at the cervical margin. The flow of noncompressible liquid is inhibited and seating of restoration is resisted [60]. The same phenomenon would have occurred in undersized post and core cementation in group 2. As the post and core approaches its final position, this space becomes smaller. Consequently, the casting of the external part complicates the proper seating of the post and core as shown in the cementation step. To alleviate this phenomenon, several methods were attempted to reduce the marginal discrepancy of the crown. Internal carving of wax patterns before casting [63], internal grinding of castings [64] venting, vibration during cementation [65], limiting the amount and site of cement placement [66], and adding a layer of die-spacer at the axioocclusal line angle [62] facilitate the drainage of excess cement and reduce the hydrostatic pressure. Additional studies to investigate the fracture resistance in presence of a core ferrule having means of cement escape are needed.

The final analysis in this study verified that fracture resistance is not associated with the cast metal post/core designed with a ferrule. The main limitation of the study is evaluating ferrule design on acrylic analogues. As a consequence, the load fracture found could not reflect the same values as for the mouth since their fracture strengths are different than teeth [67]. Thus, dynamic or fatigue behavior cannot be inferred in clinical situations until proven. However, in the literature, the use of analogues to compare the fracture resistance is valid [68–71].

Another limitation is the usage of a metallic device to hold specimens during fracture test. The fracture line and direction could have been different in the oral environment in the presence of bone and ligaments. Simulated clinical conditions might have affected the results. Further studies that simulate the oral environment are recommended.

## 6. Conclusion

Given these findings and considering the limitations of this study, it can be concluded that in presence of circumferential 2 mm of ferrule a secondary ferrule added to the cast post and core will not enhance the strength of crowned anterior teeth. A ferrule added to the cast post and core complicates the escape of the zinc phosphate during the cementation procedure.

## Figures and Tables

**Table 1:** Summary of statistical results.

Group	Load means (SD)	Displacement means (SD)	Test of normality (Lilliefors)			Mann-Whitney strength test		Kruskal-Wallis test for displacement	
			Fracture load	Displacement	Load and displacement Judgment 5%	*P* value Judgment 5%		*P *value Judgment 5%	
			*P *values Judgment 5%						
1	1038.69 (243.52)	1.36 (0.31)	0.028	0.053	Absence of normal distribution	0.7675	No significant difference	0.7470	No significant difference
2	1078.89 (335.21)	1.44 (0.58)	0.008	0.003					
